# Artificial Intelligence Meets Oral Medicine: Extending the Capabilities of Human Intelligence

**DOI:** 10.1016/j.identj.2026.109798

**Published:** 2026-08-05

**Authors:** Xinjian Ye, Tingfeng Zha, Xun Xie, Yi Xu, Li Mei, Rui Zhang, Zhiyong Wang, Qianming Chen

**Affiliations:** a Stomatology Hospital, School of Stomatology, Zhejiang University School of Medicine, Zhejiang Provincial Clinical Research Center for Oral Diseases, Key Laboratory of Oral Biomedical Research of Zhejiang Province, Cancer Center of Zhejiang University, Engineering Research Center of Oral Biomaterials and Devices of Zhejiang Province, Hangzhou, China; b Department of Developmental Biology, Harvard School of Dental Medicine, Boston, Massachusetts, USA; c Department of Oral Sciences, Faculty of Dentistry, University of Otago, Dunedin, New Zealand

**Keywords:** Artificial intelligence, Deep learning, Oral medicine, Oral disease

## Abstract

**Objectives:**

Oral medicine is an interdisciplinary field that focuses on non-odontogenic diseases. The emergence of artificial intelligence (AI) offers new opportunities to strengthen human perceptual, cognitive, and operational capacities. This review aims to provide clinicians and researchers with an explanatory, practical, and accessible overview of AI in oral medicine.

**Methods:**

This review synthesizes recent findings identified through structured online searches. Subtopics related to AI technologies in oral medicine were categorized into four areas: image recognition, decision-making support, therapy collaboration, and existing challenges. Particular emphasis was placed on oral mucosal diseases, oral cancer, and orofacial pain.

**Results:**

AI enhances clinical workflows by achieving high-accuracy lesion detection, segmentation, and classification from diverse image types. It enables data-driven diagnosis, risk stratification, and prognosis prediction. Furthermore, AI facilitates precise surgical planning, real-time guidance, and personalized postoperative management.

**Conclusions:**

AI is poised to transform Oral Medicine into an intelligence-augmented discipline. However, its full clinical integration requires overcoming challenges related to data quality, algorithmic robustness, ethical governance, and the need for robust clinical validation.

## Graphical abstract

**Figure:**
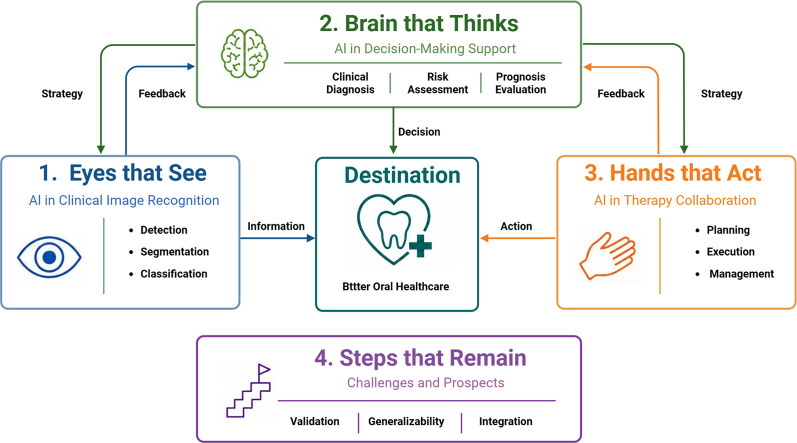

## Introduction

Oral medicine is a highly interdisciplinary field cantered on the diagnosis and management of non-odontogenic conditions, particularly oral mucosal diseases, oral cancer, and orofacial pain.^1^ These disorders often present with overlapping clinical manifestations but exhibit diverse etiologies and disease trajectories, posing significant diagnostic and therapeutic challenges. On one hand, diagnosis relies on the integration of diverse clinical data, making it highly experience-dependent and prone to inter-observer variability. On the other hand, the chronic and relapsing course of many oral diseases demands personalized risk assessment, tailored interventions, and long-term management.^2^ These complexities frequently result in bottlenecks that hinder optimal patient care and limit the efficiency of clinical practices.

With the proliferation of large-scale health datasets, artificial intelligence (AI) is catalysing transformation in modern healthcare by addressing complex clinical challenges through data-driven solutions. Its technical foundation rests on three core components: datasets, algorithms, and computational power, which interact within a structured workflow of data acquisition, intelligent analysis, and clinical output.^3^ This architecture ensures both logical consistency and adaptability across diverse clinical contexts. Oral medicine, characterized by superficial lesion presentation, abundant sample accessibility, and strong interdisciplinary integration, provides fertile ground for AI deployment. These attributes facilitate the development of an AI-powered ecosystem that supports intelligent diagnostics, risk prediction, and personalized management.^4^ Within this framework, AI is accelerating the transition from conventional treatment models toward more proactive, data-driven, and precision-oriented healthcare delivery.

Several subdomains of AI show substantial promise in oral medicine. Among them, machine learning enables models to identify patterns and make predictions from data without explicit programming.^5^ Common approaches include supervised learning for classification and regression, unsupervised learning for clustering and anomaly detection, and reinforcement learning for decision optimization. Deep learning, a subset of machine learning, leverages neural networks to process complex datasets and has been widely applied to image recognition and lesion segmentation, particularly through convolutional neural networks (CNNs).^6^ More recently, AI-powered large language models (LLMs), such as ChatGPT and DeepSeek, have emerged as transformative tools capable of processing unstructured clinical data and generating medically relevant insights.^7^ Unlike traditional models that rely on structured inputs, LLMs interpret free-text information, enabling integration of multimodal clinical inputs and supporting explainable, text-based decision-making in oral healthcare.

Undoubtedly, while AI was initially developed to mimic human cognition, it has since evolved to surpass specific limitations of human intelligence in narrowly defined tasks. Its greatest strength is increasingly recognized not in replacing human expertise, but in collaborating alongside it to advance oral healthcare. By analysing high-dimensional data and augmenting human reasoning capacities, AI enables clinicians to see more clearly, think more critically, and act more precisely. This critical review highlights current clinical applications of AI in oral medicine and explores its emerging role in reshaping diagnostic and therapeutic paradigms.

In this review, a structured literature search was conducted in major electronic databases, including PubMed, Web of Science, and Scopus, covering studies published up to January 2026. The search strategy combined terms related to artificial intelligence and oral medicine, including ('artificial intelligence' OR 'machine learning' OR 'deep learning' OR 'large language models') AND ('oral medicine' OR 'oral disease' OR 'oral cancer') AND ('diagnosis' OR 'risk assessment' OR 'prognosis' OR 'treatment'). Retrieved records were screened for relevance to the scope of this review, and studies were selected on the basis of methodological relevance, topical representativeness, and clinical significance, with particular emphasis on recent and clinically informative publications (Table S1).

## Eyes that see: AI in clinical image recognition

**Fig. 1:**
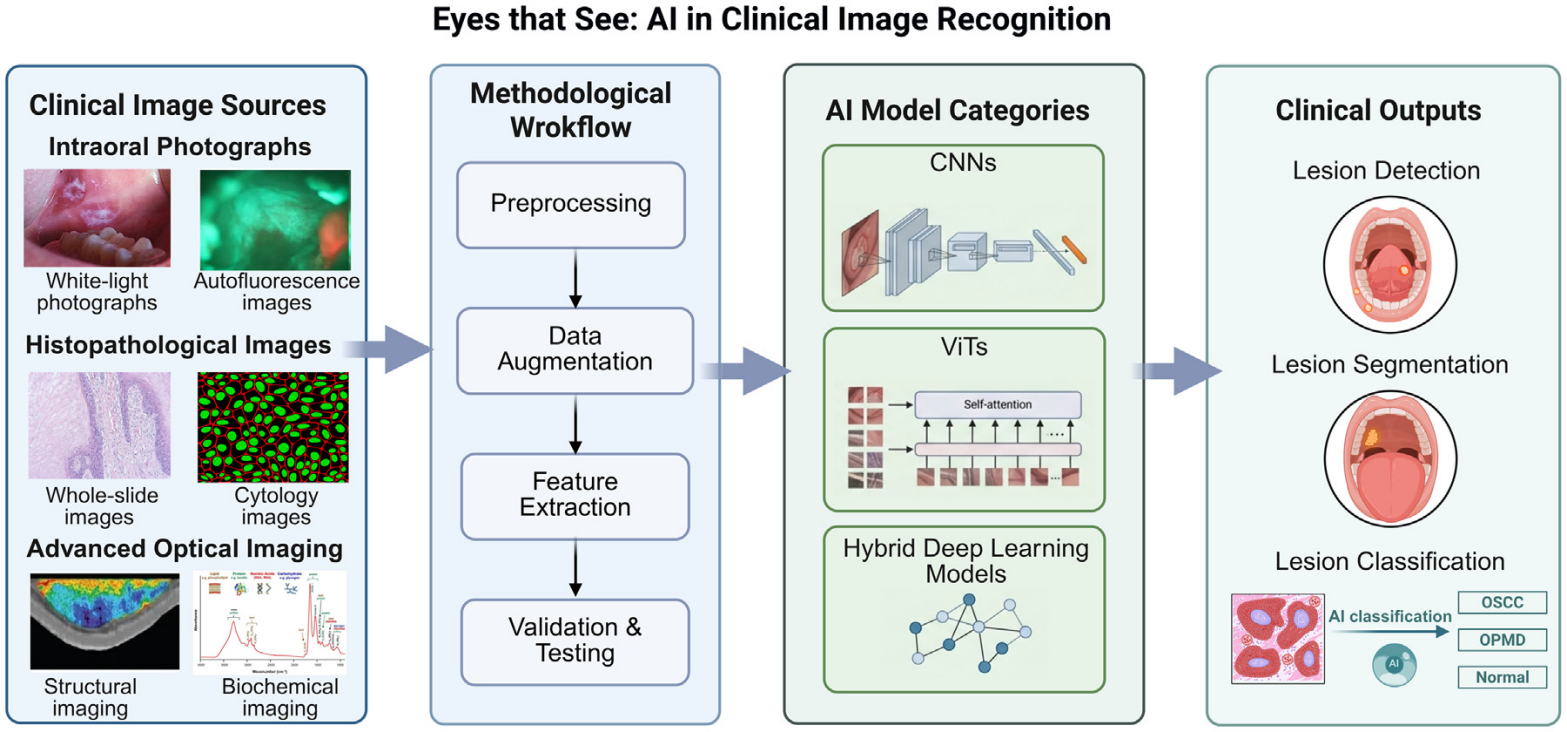
AI models enable precise and scalable detection of oral lesions by learning disease-specific visual patterns from clinical images. AI, artificial intelligence; CNNs, convolutional neural networks; ViTs, vision transformers.

Effective recognition of oral lesions relies on precise spatial interpretation of clinical images to identify lesion presence, delineate affected regions, and assess disease severity. However, subtle visual cues and significant inter-class similarity often challenge human interpretation, leading to the potential of oversight or misclassification. AI models, such as CNNs and vision transformers, overcome these challenges by learning disease-specific visual patterns from annotated data, enabling the transformation of raw images into clinically meaningful outputs for more accurate, reproducible, and scalable image recognition.^8^ This section outlines how AI systems, equipped with a 'sharp eye', are applied across diverse imaging modalities to enhance lesion identification in oral medicine (Figure 1).

### Intraoral photographs

Oral lesions are superficial, observable, and easily accessible, making them well-suited for AI-based photographic imaging analysis. Intraoral photographs serve as the primary visual reference for screening, documentation, and monitoring, capturing essential features such as colour, texture, location, and morphology. These standardized, high-quality visual inputs provide an ideal foundation for AI-driven approaches, which can preprocess raw images to enable automated lesion detection, segmentation, and classification.^9^

Lesion detection in intraoral photographs is challenged by variability in image quality due to variations in lighting, angle, resolution, and patient positioning. To ensure reliable performance, AI-based workflows aim to standardize image inputs and improve recognition accuracy. Wang et al applied multiscale Retinex algorithms to enhance intraoral image quality, which significantly improved the sensitivity and accuracy of AI-based detection of complex oral potentially malignant disorders (OPMDs) lesions.^10^ A meta-analysis of 34 studies found that AI achieved 74% to 100% accuracy in detecting oral mucosal lesions from photographs. The pooled diagnostic odds ratio was 155 for OPMDs and 114 for cancerous lesions.^11^ Similarly, Patel et al proposed an attention-guided CNN that focused on detecting common oral lesions from intraoral images. By integrating ground truth segmentation masks into the training process, their model aligned attention maps with lesion regions, improving localization accuracy and minimizing the impact of dataset bias.^12^

Manual segmentation of oral lesions is often time-consuming and subjective, especially in cases with vague lesion borders or overlapping tissue structures. AI-based segmentation models offer a scalable solution by automatically delineating lesion boundaries with improved precision and reproducibility. In this context, our previous study developed a SegFormer-based semantic segmentation model capable of automatically delineating common oral mucosal lesions in Intraoral Photographs. The model outperformed classical approaches, achieving a Dice score of 0.71 and a mean intersection over union of 0.79.^13^ Similarly, Alzahrani et al proposed a deep learning-structured approach incorporating vision intelligence for the segmentation of oral squamous cell carcinoma (OSCC) lesions, achieving a superior accuracy of 99% on medical images.^14^

Reliable classification of oral lesions remains a challenge due to substantial inter-class resemblance and intra-class variability. From raw pixels to clinical interpretation, AI models trained on disease-specific visual patterns enable accurate and standardized diagnostic classification. For instance, multiple CNN architectures have been developed to classify recurrent aphthous ulcers (RAU) based on clinical photographs. Among them, ResNet50 showed the best performance, achieving a precision of 93%, a recall of 92%, an F1 score of 92%, a specificity of 96%, a sensitivity of 92%, and an area under the receiver operating characteristic curve (AUC) of 0.99.^15^ Similarly, Wu et al developed a CNN-based smartphone application for lupus erythematosus, demonstrating robust differentiation across six subtypes, with an AUC of 0.97, accuracy of 91%, sensitivity of 93%, and specificity of 90%.^16^ Another study employed vision transformer models for the multi-class classification of OPMDs and OSCC, reporting F1 scores between 0.80 and 0.85, and AUCs ranging from 0.94 to 0.97.^17^

### Histopathological images

Histopathological slides, typically stained with haematoxylin and eosin, provide detailed visualization of cellular and subcellular structures and remain the gold standard for the definitive diagnosis of oral lesions. These images reveal morphological features such as nuclear pleomorphism, mitotic activity, tissue disorganization, and invasion depth, which are essential for dysplasia grading and malignancy identification. AI models are increasingly employed to automate histological pattern recognition, assisting pathologists in performing more refined and standardized diagnoses.^18^

AI models demonstrate pathologist-level performance in histopathological image analysis, with the capacity to identify subtle cellular and molecular features for precise diagnosis. For example, Wu et al developed a graph learning-based model for the automated detection of Sjögren’s syndrome in salivary gland biopsies, achieving an AUC of 0.80, along with 98% sensitivity and 94% accuracy.^19^ Dharani et al proposed a deep learning framework for the segmentation of OSCC in histological slides, reporting an accuracy of 99%, sensitivity of 99%, and specificity of 97%.^20^ Extending these advances, an interpretable foundation model was developed to infer human papillomavirus status and head and neck squamous cell carcinoma (HNSCC) subtypes directly from histopathological images.^21^

Oral epithelial dysplasia (OED) is a premalignant condition characterized by architectural disruption and cytological atypia. Histopathological grading of OED is crucial for diagnosis, but subject to inter-observer variability. AI models can improve consistency by learning morphological patterns from annotated histological data, enabling more standardized and reproducible grading.^22^ Liu et al developed a CNN-based model to assist in grading OED on whole-slide images and highlight suspected high-grade regions, achieving an accuracy of 93% and an F1-score of 91%.^23^ Hadilou et al employed a vision transformer-based model to classify OED in histopathological images, outperforming conventional CNNs in both three-class and four-class grading tasks.^24^ Expanding on these work, our previous study integrated feature detection techniques with a multiclass logistic regression framework, resulting in a comprehensive system that achieved an accuracy of 87% and an AUC of 0.67 in predicting pathological features and grading OED.^25^

### Advanced optical imaging

Advanced optical modalities, broadly classified into structural and biochemical imaging, enable visualization of subtle morphological features and molecular-level alterations often undetectable by conventional white-light imaging. Characterized by their non-invasive, high-resolution, and label-free nature, these techniques are particularly valuable for early lesion detection, functional tissue assessment, and real-time clinical evaluation. When integrated with AI, they further enhance diagnostic accuracy through automated interpretation and multimodal data integration, leading to more consistent, objective, and precise clinical decision-making.^26^

Structural imaging captures spatial variations in optical properties to visualize tissue morphology, with optical coherence tomography (OCT) serving as a key modality that employs low-coherence interferometry to produce high-resolution, cross-sectional images of tissue microarchitecture.^27^ A systematic review of nine studies involving 860 cases demonstrated that AI-assisted OCT interpretation consistently outperformed both clinician assessment and conventional algorithms, particularly in early cancer detection and surgical margin delineation.^28^ Specifically, a multi-level deep residual learning framework was developed for OSCC diagnosis using OCT images, achieving superior sensitivity, specificity, and accuracy at both image and patch levels.^29^ Zheng et al proposed a systematic workflow that integrates rapid OCT angiography data acquisition with deep learning-based image reconstruction, enabling high-quality, quantitative visualization of vascular microcirculation in oral diseases.^30^

Biochemical imaging characterizes the molecular composition of tissues and detects pathological changes by capturing spectroscopically derived signals. AI shows strong potential in analysing spectroscopic imaging data across various modalities. A scoping review of fourteen studies summarized the use of infrared spectroscopy combined with machine learning in head and neck cancer research, reporting sensitivity and specificity up to 100%, accuracy up to 96%, and an AUC of 0.99.^31^ Another study combined sub-diffuse reflectance spectroscopy with machine learning to classify oral lichen planus (OLP), oral leukoplakia (OLK), OSCC, and normal tissue, achieving an accuracy of 0.83, sensitivity of 0.85, and specificity of 0.93.^32^ Additionally, surface-enhanced Raman spectroscopy, optimized with gold nanoparticle-based substrates, has also been combined with deep learning to accurately classify oral cancer stages using saliva samples.^33^ For fluorescence spectroscopy, an AI-integrated portable device has been developed for real-time assessment of oral mucosal lesions, enabling reliable differentiation among normal tissue, dysplasia, and OSCC.^34^ Similarly, the combination of autofluorescence and brightfield imaging within an AI-driven multimodal fusion model has been shown to significantly improve cytological classification and cancer detection in brush biopsy specimens.^35^

## Brain that thinks: AI in decision-making support

**Fig. 2:**
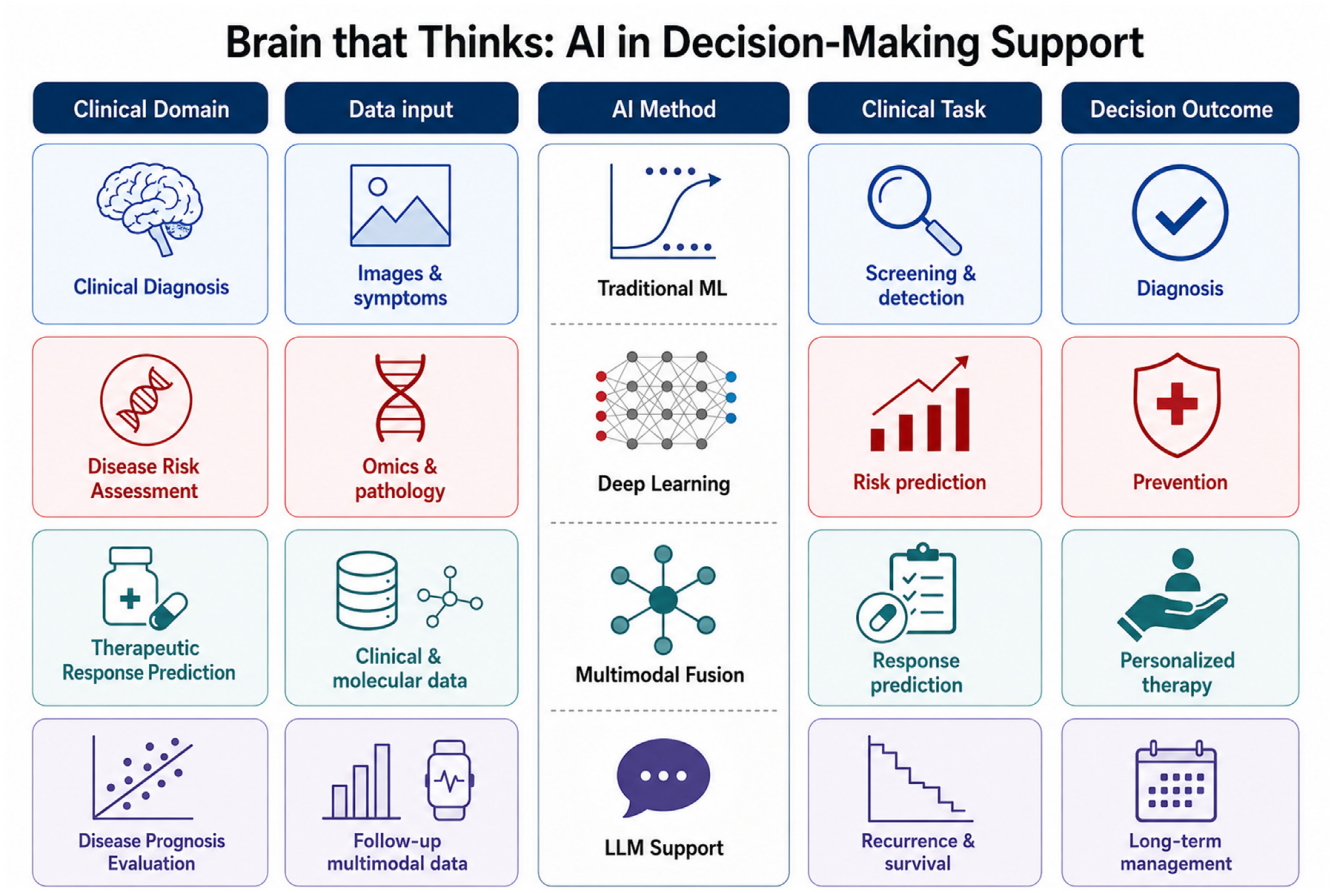
AI integrates multimodal clinical data to enhance diagnostic accuracy and enable predictive, personalized decision-making in oral medicine. AI, artificial intelligence.

In traditional clinical workflows, decision-making hinges on expert interpretation of multifaceted data, a process inherently constrained by subjectivity and variability. AI models, trained on large-scale, high-quality datasets, not only replicate expert-level reasoning by identifying clinically meaningful patterns, but also provide predictive foresight into disease progression and treatment response. By shifting clinical practice from retrospective assessment to proactive, personalized intervention, these systems enhance both diagnostic precision and therapeutic planning. This section explores how AI, functioning as a 'clever brain', redefines diagnostic accuracy and empowers anticipatory care across the oral healthcare continuum (Figure 2).

### Clinical diagnosis

Accurate clinical diagnosis is the cornerstone of effective oral disease management. AI systems enhance diagnostic reliability by analysing complex visual and clinical data, supporting standardized identification, differentiation, and classification of oral diseases. Recent advances in natural language processing further move beyond pixel-based perception to multimodal sources that better reflect real-world clinical reasoning.^7^

Reliable differentiation of oral mucosal diseases remains clinically challenging due to overlapping clinical presentations across lesion types. AI systems offer promising capabilities for efficiently distinguishing among fundamental patterns of mucosal pathology. Zhan et al reported that ChatGPT can interpret oral mucosal disease photographs to generate structured clinical reports in complex, multimodal diagnostic tasks, producing fine-grained items including location, shape, number, size, clinical manifestation, lesion border, and diagnosis.^36^ Similarly, a retrospective analysis suggested that ChatGPT can moderately interpret histopathological descriptions of oral and maxillofacial lesions, although performance varies with lesion subtype.^37^ Building on this, a recent study demonstrated that ChatGPT achieved high sensitivity in the differential diagnosis of oral mucosal lesions based on lesion descriptions.^38^ Another study evaluated the diagnostic accuracy of chatbot systems in clinical case scenarios related to oral pathologies. The DeepSeek model demonstrated superior performance compared to ChatGPT, although both systems require further optimization for clinical application.^39^

Many oral mucosal diseases carry a risk of malignant transformation, making early detection of precancerous lesions essential for timely intervention and improved patient outcomes. AI-powered smartphone applications provide a practical solution for oral lesion screening by providing real-time, user-friendly, and remote diagnosis. A meta-analysis of 17 studies reported that AI-based models achieved high diagnostic performance, with a sensitivity of 90%, specificity of 89%, and overall prediction accuracy of 90% for the detection and classification of OPMDs and OSCC.^40^ CNNs have been shown to effectively analyse smartphone-captured oral photographs for early OSCC detection, particularly in identifying suspicious lesions at anatomically relevant sites.^41^ Building on this potential, a YOLO-based CNN was integrated into a mobile application for the detection of OLP, OLK, and OSCC, achieving a recall of 85%, precision of 87%, and specificity of 95%.^42^ Additionally, our study proposed a two-stage deep learning method, achieving 89.9% F1-score for OPMD distinction and 92.2% for OPMD subtype classification, which significantly outperforms clinicians.^43^ In parallel, a bimodal smartphone device combining fluorescence imaging and spectroscopy demonstrated strong performance in oral cancer detection and biopsy guidance, with an accuracy of 97%, sensitivity of 95%, and specificity of 97%.^44^

Orofacial pain includes a range of conditions with overlapping features affecting the face and oral cavity. A recent study developed a machine learning-compatible documentation system for real-time diagnosis of orofacial pain, demonstrating high concordance with expert assessments.^45^ In terms of differential diagnosis, LLMs have been shown to accurately identify burning mouth syndrome (BMS) and distinguish it from other orofacial pain conditions, achieving diagnostic accuracies between 89% and 99%.^46^ Similarly, a ChatGPT-based clinical decision support system for orofacial pain demonstrated the ability to improve diagnostic accuracy and generate structured differential diagnoses within 15 minutes.^47^ Facial expressions, as key nonverbal indicators of pain, can be analysed by AI systems to enhance objective pain evaluation. A video-based spatiotemporal attention framework demonstrated strong performance in multi-level pain detection while remaining independent of verbal input.^48^ To improve privacy and reduce invasiveness, an AI-based 3D facial landmarks was introduced, enabling accurate facial analysis without raw image data and enhancing security and feasibility in clinical applications.^49^

### Disease risk assessment

Moving beyond reactive care, disease risk screening embraces a preventive paradigm by identifying individuals with elevated susceptibility before clinical onset. At both population and individual levels, AI enables precise risk stratification, advancing early intervention and cross-disciplinary prevention strategies.^50^

OED poses a high risk of malignant transformation, making accurate risk stratification essential for informed clinical management and biopsy decision-making. In response, AI-based models have been developed to support malignant risk prediction.^51^ Adeoye et al proposed a deep learning system that estimates dysplasia probability and malignant transformation in OLK, achieving an AUC of 0.88 and an accuracy of 82% for OED calibration and discrimination.^52^ An AI-based oral dysplasia network was developed to classify OED and predict its transformation risk from whole-slide histopathological images, yielding an F1 score of 0.96 and an AUC of 0.73.^53^ In related research, a self-attention neural network applied to real-world data outperformed traditional machine learning and epidemiologic models in predicting malignant transformation in OPMDs.^54^ Extending this approach, Shephard et al developed an AI algorithm that emulates histological assessment and identified intraepithelial lymphocyte density as a potential prognostic indicator of OED progression.^55^

Leveraging high-resolution inference across large-scale datasets, AI-driven integration of multi-omics data further advances OSCC risk assessment, facilitating more precise and personalized prevention strategies. Machine learning models have demonstrated strong performance across diverse omics platforms. Applied to metabolomic data, they identified key biomarkers that distinguished OSCC patients from healthy controls, achieving 77% accuracy and an AUC of 0.93.^56^ Similarly, models trained on cancer-associated microRNA expression reached 89% accuracy and an AUC of 0.90 for OSCC detection and prediction.^57^ In transcriptomic analyses, AI-identified differentially expressed genes highlighted strong prognosis relevance, with an average classification accuracy of 84% across various algorithms.^58^ Extending to the oral microbiome, metagenomic and 16S rRNA sequencing data revealed microbial-metabolic risk factors associated with HNSCC progression, yielding AUCs between 0.78 and 0.89.^59^

Notably, the oral cavity is closely interconnected with systemic health, serving as both a mirror and a mediator of systemic conditions through shared microbial, inflammatory, and immunological pathways.^60^ In this increasingly interdisciplinary landscape, AI-based models have advanced understanding of the oral-systemic axis by enabling bidirectional risk prediction. On one hand, AI has been applied to infer systemic disease risk based on oral indicators. For instance, machine learning models incorporating tongue image features and oral-gut microbiota achieved 79% accuracy with an AUC of 0.87 in predicting prediabetes and type 2 diabetes.^61^ Similarly, an AI-assisted visual risk prediction system using salivary microbial variation demonstrated strong performance in estimating the risk of persistent pulmonary nodules, with an AUC of 0.88 and an F1 score of 0.78.^62^ On the other hand, systemic parameters have been utilized to assess oral disease risk. Machine learning models predicting diabetic oral ulceration based on oral examination data reached an AUC of 0.91 and 95% accuracy.^63^ Likewise, a cross-sectional study utilizing peripheral blood biomarkers for the classification and prediction of oral mucosal diseases, including RAU, OLP, BMS, and atrophic glossitis, reported an AUC of 0.89 and an accuracy of 95%.^64^ A recent study proposed a fusion machine learning model that integrated tongue dorsum features and microbiome profiles to identify coronary artery disease, achieving an AUC of 0.86, an accuracy of 70%, and a sensitivity of 90%.^65^

### Disease prognosis evaluation

Prognostic evaluation focuses on anticipating disease trajectories after diagnosis to inform treatment planning and long-term management, particularly in malignant conditions. By integrating multimodal data, AI models enhance the prediction of disease progression and clinical outcomes.

This predictive potential is exemplified in several AI-driven models for oral cancer prognosis. Mei et al evaluated clinical, pathological, and haematological data from 1163 OSCC patients to develop machine learning models for tumour progression prediction, with logistic regression demonstrating the highest performance.^66^ A fuzzy deep learning-based model using clinical and pathological data achieved a 97% accuracy and a perfect AUC in estimating the survival time of OSCC patients.^67^ In another study, machine learning applied to single-cell sequencing data characterized the immune micro-environment and established an immune infiltration prediction model, offering reliable support for tumour prognosis evaluation.^68^

Multimodal fusion further enhances predictive modelling by capturing complementary imaging and molecular features. For example, combining lipid metabolite profiling with magnetic resonance imaging (MRI)-based transformer and radiomics models improved tumour stage prediction in OSCC, achieving an AUC of 0.87.^69^ Similarly, a transformer-based multimodal framework that integrates radiological and pathological data outperformed unimodal models in predicting disease-free survival and tumour grade in HPV-associated oropharyngeal squamous cell carcinoma.^70^ Moreover, a deep radiomics model incorporating OCT imaging and peripheral immune indicators demonstrated strong prognostic accuracy and inflammatory risk stratification in OSCC.^71^

Prediction of recurrence and metastasis is another key component of prognostic evaluation in oral cancer. In this context, a multilayer perceptron model developed for postoperative recurrence prediction achieved an accuracy of 84% and an AUC of 0.89 using comprehensive clinical inputs.^72^ Likewise, a population-based study of 1320 patients demonstrated that machine learning models incorporating patient-reported outcomes achieved moderate accuracy in predicting recurrence in HNSCC.^73^ For metastasis assessment, radiomics-based models using multimodal MRI have demonstrated high accuracy and stability in detecting cervical lymph node metastasis in tongue squamous cell carcinoma.^74^ Building on this progress, a recurrence prediction model that integrated AI-extracted histopathological features with manually assessed clinicopathological data achieved an outstanding AUC of 0.99.^75^

### Therapeutic response prediction

Personalized treatment requires accurate prediction of individual patient responses to therapeutic interventions, moving beyond one-size-fits-all approaches to evidence-based, tailored care strategies. AI-driven predictive models integrate patient-specific clinical, genetic, and phenotypic data to forecast treatment efficacy and adverse reactions, enabling clinicians to select optimal therapeutic protocols and improve patient outcomes.^76^

The treatment of oral mucosal diseases involves a combination of etiological, physical, and surgical therapies. Although AI-assisted medication management remains limited, it holds considerable promise for developing personalized treatment strategies tailored to individual patient profiles.^77^ In cases of atypical OLK, AI can synthesize clinical and pathological data to support surgical decision-making, thereby improving treatment precision and reducing unnecessary surgeries.^51^ Photodynamic therapy (PDT) is a minimally invasive modality that employs light-activated photosensitizers to induce targeted cytotoxicity in dysplastic tissues. In our previous study, a deep learning model achieved 84% accuracy and an AUC of 0.88 in predicting short-term treatment response and recurrence risk in OLK patients.^78^ Moreover, an AI-based cytology platform utilizing DNA aneuploidy analysis allows real-time monitoring of PDT efficacy by identifying abnormal cells in brush biopsy samples.^79^

Treatment modalities for oral cancer include surgery, radiotherapy, chemotherapy, and emerging immunotherapies. AI-driven predictive models are increasingly applied to optimize treatment strategies by forecasting therapeutic responses and informing complex clinical decisions. In surgical oncology, machine learning has been applied to stratify patients with locally recurrent OSCC into distinct risk categories, identifying those most likely to benefit from salvage surgery.^80^ For chemoradiotherapy, a large-scale study involving 33,526 HNSCC patients showed that AI-based survival models effectively identified candidates most likely to respond favourably to combined therapy.^81^ Furthermore, Alabi et al achieved 86% accuracy in predicting overall survival in advanced-stage tongue OSCC patients treated with surgery and adjuvant radiotherapy.^82^ Similarly, Song et al utilized CT-derived radiomic signatures to estimate survival outcomes and guide chemotherapy de-escalation in HPV-associated oropharyngeal cancer.^83^

Predictive modelling is a pivotal tool for addressing the heterogeneity of immunotherapy response. A neural network model based on CT radiomics demonstrates exceptional efficacy in predicting immunotherapy response for OSCC, achieving an AUC of 0.86 with 83% accuracy, sensitivity, and specificity.^84^ Furthermore, an MRI-derived deep learning scoring system has also attained significant outcomes, yielding an AUC of 0.74 in predicting both PD-L1 expression and durable clinical benefit.^85^ Another study integrated intratumoral and peritumoral MRI features in OSCC patients, achieving an AUC of 0.91 for predicting neoadjuvant chemoimmunotherapy efficacy.^86^ By probing the molecular circuitry of oral cancer, AI is identifying genetic cues that drive innovation in precision immunotherapy. Machine learning combined with multi-omics analysis has successfully identified key drug-resistance genes and their ligands in OSCC patients.^87^ while semi-supervised adversarial domain adaptation techniques accurately predict single-cell drug responses and identify drug-resistant tumour subpopulations from transcriptomic data.^88^

Managing complications represents another critical aspect of treatment response. AI enhances postoperative care by predicting potential complications and evaluating risk profiles, thereby enabling more precise and targeted preventive strategies. For example, one model for radiation-induced oral mucositis in HNC patients achieved an AUC of 0.92, with 90% accuracy, 83% sensitivity, and 100% precision.^89^ In another effort, four machine learning approaches predicted postoperative malnutrition in oral cancer patients, with XGBoost leading the pack.^90^ In addition, a random forest algorithm, trained on 412 oral cancer cases, effectively anticipated hypotension after tumour removal and flap reconstruction.^91^ CNNs, meanwhile, track recovery via imaging, spotting infections or issues early to enable prompt action.^92^

## Hands that act: AI in therapy collaboration

**Fig. 3:**
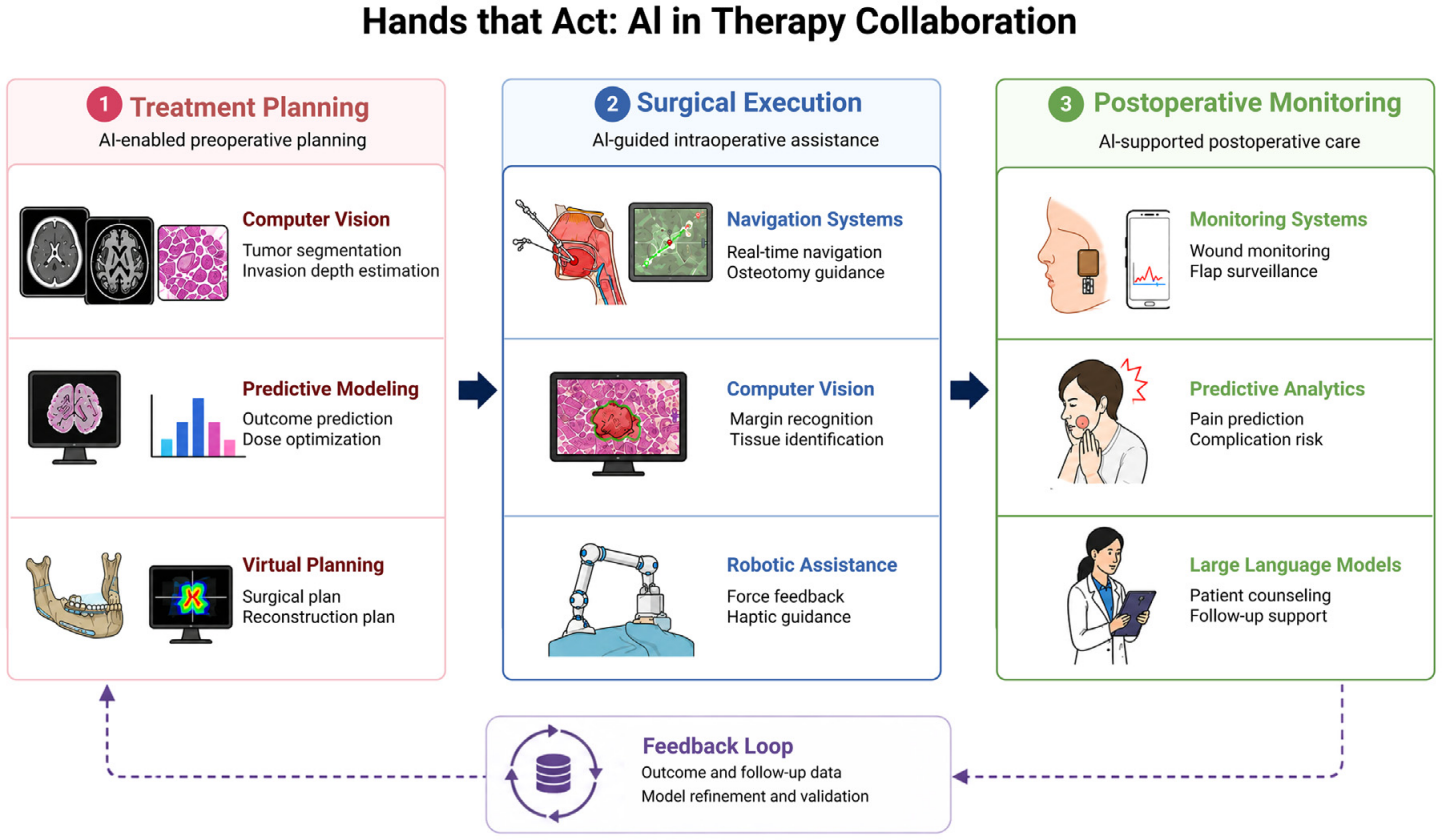
AI-assisted technologies enhance surgical precision in oral cancer by supporting intelligent preoperative planning, real-time intraoperative navigation, and integrated postoperative management. AI, artificial intelligence. AI, artificial intelligence; LLM, large language model; ML, machine learning.

Surgical resection remains the cornerstone of curative therapy for oral cancer, yet it is challenged by anatomical complexity, operator-dependent variability, and limited operative adaptability. AI-powered technologies are redefining this surgical paradigm by enabling intelligent preoperative planning, real-time intraoperative navigation, and integrated postoperative management. These systems convert static imaging and clinical data into actionable insights. This section illustrates how AI, functioning as a 'skilled hand', elevates surgical collaboration from manual execution to intelligent orchestration, steering the shift toward data-driven, personalized cancer care (Figure 3).

### Treatment planning

Surgical success in oral oncology depends on meticulous planning and coordinated execution across all stages—from preoperative design and intraoperative strategy to reconstruction and adjuvant therapy. Despite advances in technique, variability in planning remains a barrier to optimal outcomes. AI offers a paradigm shift by integrating multimodal data to guide strategy, real-time guideline, and personalize postoperative care with greater precision.

At its core, AI informs incision design and operative strategy, enabling more individualized and effective interventions. For example, deep learning algorithms can provide real-time detection of three-dimensional tumour margins and predict invasion depth with errors as small as 0.4 mm prior to resection.^93^ U-Net architectures applied to contrast-enhanced MRI achieve tumour segmentation with mean Dice coefficients of 0.87, markedly reducing inter-observer variability.^94^ Building on this progress, AI-enhanced virtual surgical planning (VSP) has evolved from static visualization tools into intelligent, adaptive platforms capable of real-time guideline. Hagen et al developed an AI-driven VSP system that produces high-quality mandibular reconstruction plans for tumour cases within 20 minutes, significantly streamlining the surgical workflow.^95^

Beyond tumour excision, definitive oncologic care requires reconstructive strategies that restore both facial aesthetics and functional integrity. AI facilitates this process through advanced anatomical modelling, translating complex imaging data into surgical blueprints for accurate reconstruction.^96^ For skeletal repair, automated systems achieve mandibular reconstruction with sub-2 mm accuracy, and CT-based platforms can generate bone structures automatically, providing ready datasets for 3D printing-assisted procedures.^97^ Soft tissue restoration has also advanced, with AI optimizing bioprinting parameters for greater efficiency and precision. In facial repair specifically, AI analyses of defect morphology and donor tissue availability offer customized surgical options that balance function and aesthetics.^98^ Moreover, predictive models derive soft-tissue profiles from underlying craniofacial bones, while hierarchical representation networks reconstruct 3D facial features from standard photographs with high accuracy, guiding surgeons in preoperative planning.^99^

Radiotherapy is another scalpelagainst oral cancer, where success hinges on precise dose delivery, target localization, and protocol adherence. In treatment planning, AI automates intensity-modulated radiotherapy plans in under 15 minutes, enhancing dose precision and cutting preparation time.^100^ For target segmentation, a CNN method segmented organs at risk in 664 HNSCC patients, yielding Dice similarity coefficients above 0.70.^101^ A deep learning model, seamlessly integrated into the commercial system, shows no significant difference from manual planning in key metrics, such as the average dose to the parotid and oral cavity or the maximum dose to the brainstem and spinal cord.^102^ During implementation, online adaptive radiation therapy adjusts plans dynamically for tumour and anatomical shifts. When paired with deep learning, it achieves substantial dose sparing—up to 5.47 Gy.^103^

### Surgical execution

Even the most carefully planned surgeries encounter intraoperative uncertainties that demand rapid and adaptive decision-making. By integrating continuously updated navigation systems, real-time margin assessment, and robotic assistance, AI is redefining surgical boundaries and enabling a new era of submillimetre precision.

Operative navigation, often likened to a 'surgical GPS', enhances lesion localization to reduce collateral tissue damage and complications. With AI integration, these systems now deliver submillimetre spatial accuracy. For example, neural networks applied to CT point clouds and optical tracking data reduced localization errors from about 2 mm to less than 1 mm.^104^ Similarly, AI-driven navigation enables real-time osteotomy adjustments, lowering angular deviations to 1.08 ± 0.66°compared with 2.32 ± 0.71°in traditional systems.^105^ A deep learning model automatically segment loose connective tissue fibres to identify safe dissection planes,^106^ while AI-enhanced fluorescence lifetime imaging generates tumour probability heatmaps that support more precise resections with fewer residual cells.^107^

The surgical dilemma of 'cutting too little or too much' has challenged surgeons for decades. While frozen section analysis remains subjective, AI-powered tools offer instant and objective guidance on tumour margins. For example, a study examined 1329 elastic scattering spectroscopy measurements from 104 patients using leave-one-patient-out cross-validation, achieving 94% sensitivity, 87% specificity, and an AUC of 0.95 for patient-level intraoperative margin assessment.^108^ Likewise, CNNs applied to hyperspectral imaging datasets enable real-time discrimination between neoplastic and healthy tissues with 86% accuracy.^109^ Marsden et al successfully distinguished healthy from cancerous tissues by combining fluorescence lifetime imaging and machine learning models, including SVM, random forests, and CNNs.^107^ Moreover, surpassing conventional intraoperative assessment, an AI generative model achieved 87% accuracy in diagnosing tumour versus normal muscle by converting Raman images into virtual haematoxylin-eosin histopathology.^110^

Robotic surgery, when combined with AI, represents another transformative step. Intelligent automation brings precision and consistency, while AI-enabled haptic feedback restores the tactile sense often lost in minimally invasive procedures. Embedded force sensors within robotic instruments capture physical interaction data, which are then translated into real-time tactile feedback through AI algorithms and specialized user interfaces.^111^ Beyond direct sensing, machine learning techniques correlate visual cues of tissue deformation with predicted tactile responses, thus establishing 'virtual haptics'. For instance, a neural network–based inverse dynamics identification method estimated internal torque from joint kinematics, allowing derivation of external forces with mean errors <10%.^112^ Similarly, an end-to-end deep learning framework trained on Da Vinci robot inputs achieved force estimation accuracy within 1 to 2 N across a 10 N range, providing clinically feasible haptic feedback solutions.^113^

### Postoperative management

Effective postoperative care is indispensable to surgical success, yet it remains time- and labour-intensive. Acting as an intelligent partner rather than a mere tool, AI can assist clinicians by automating perioperative monitoring and supporting long-term patient counselling and follow-up.

Wound management is a cornerstone of perioperative care, demanding both vigilance and precision. Smart wound dressings embedded with biosensors can continuously track temperature, pH, and moisture, transmitting real-time data to AI systems that flag early signs of infection or perfusion compromise.^114^ For free flap monitoring in head and neck cancer patients, AI-integrated wearable Doppler devices can detect vascular compromise hours before clinical symptoms emerge, enabling timely intervention and flap salvage.^115^ Proactively, patients can upload wound images via smartphone platforms, where computer vision algorithms automatically analyse healing trajectories, detect subtle morphological changes, and differentiate normal postoperative recovery from early complications.^116^

Pain management evolves from simple symptom relief to a precision discipline that shapes postoperative recovery. Machine learning integrates patient characteristics, genetic profiles, and procedural details to forecast pain trajectories and individualize analgesic regimens, reducing both undertreatment and opioid overuse.^117^ Salama et al developed models predicting acute pain, opioid requirements, and treatment outcomes in oropharyngeal cancer patients receiving radiotherapy; these models accurately anticipated pain intensity, daily morphine doses, and therapeutic response.^118^ Moreover, NLP has identified linguistic markers of inadequate pain control and potential medication misuse, enabling more responsible prescribing practices.^119^

Structured follow-up is essential after oral cancer surgery, demanding both high patient adherence and coordinated care systems. AI platforms can bridge this gap by delivering continuous patient education, long-term emotional support, timely professional consultations, and integrated medical record management. In clinical follow-up scenarios, ChatGPT has demonstrated not only high accuracy in knowledge delivery but also strong empathetic communication, supporting both informational and psychological needs.^120^ Personalized learning systems further tailor educational content to patient literacy levels, learning preferences, and procedure-specific requirements, ensuring critical information is communicated effectively and at appropriate time points.^116^ Moreover, AI-based home rehabilitation platforms empower patients with HNSCC to actively manage their recovery, improve functional outcomes, and enhance quality of life through data-driven self-care strategies.^121^

## Steps that remain: challenges and prospects

**Fig. 4:**
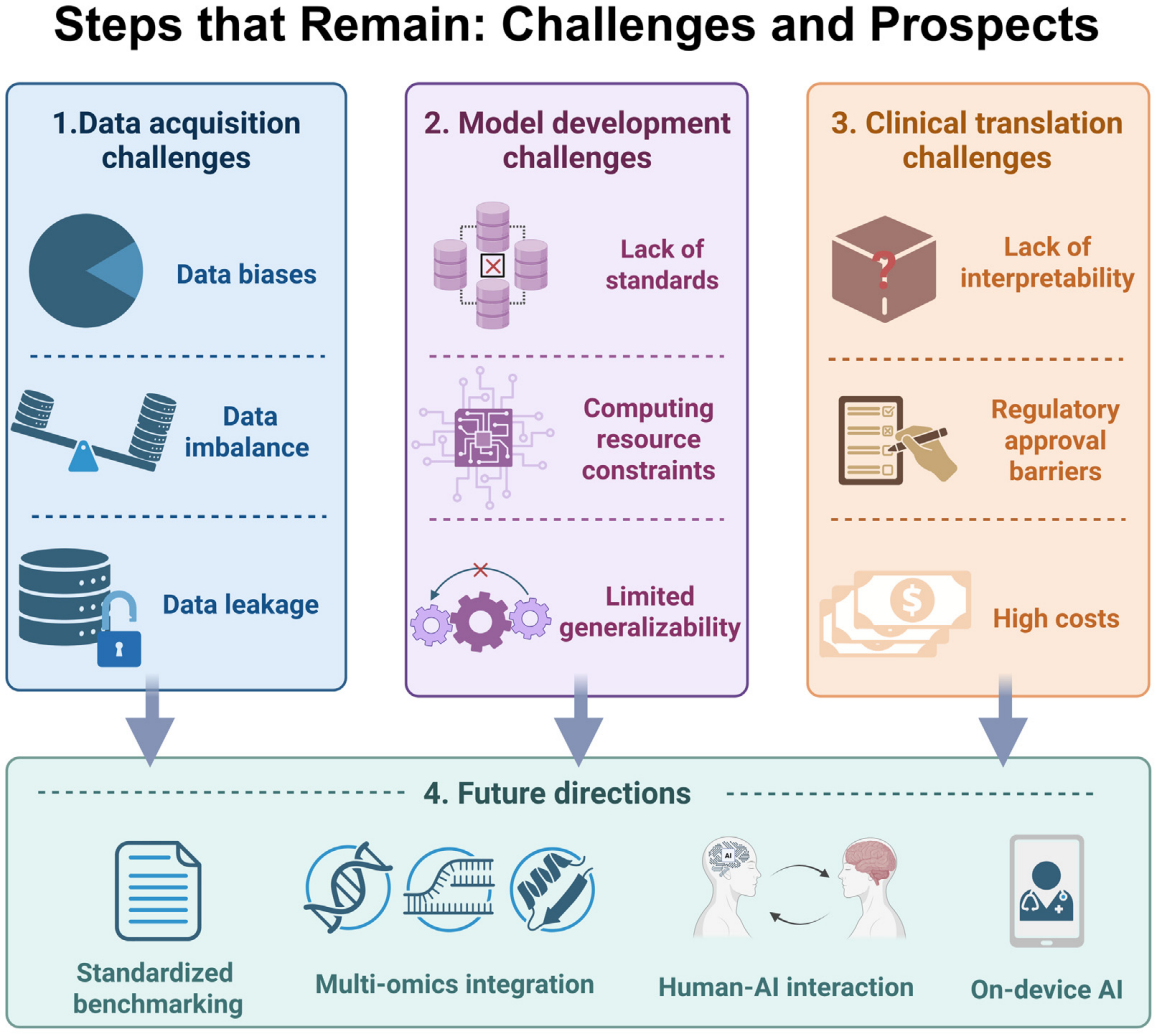
Ongoing challenges in data acquisition, analytical intelligence, and clinical validation continue to hinder the transition of AI in oral medicine from experimental development to clinical implementation. AI, artificial intelligence.

Although AI has learned to see with precision, think with insight, and act with skill in oral medicine, its journey toward a trusted clinical partner remains incomplete. Recent comprehensive overviews of AI in dentistry have provided a broader framework for understanding both the transformative potential and the current limitations of AI-driven healthcare.^122^,^123^ Critical steps remain—both universal to medical AI and specific to oral healthcare—before these intelligent eyes, brains, and hands can move beyond experimental prototypes to become indispensable allies in routine clinical practice (Figure 4).

### Data acquisition

The effective application of AI depends on large-scale, high-quality datasets that are indispensable for model training and inference. In oral medicine, however, data resources remain comparatively limited, with marked imbalances across subfields: databases for oral cancers are relatively well developed, whereas those for oral mucosal diseases, rare conditions, and special populations are severely underrepresented (Table S2). Although several deidentified public databases are now available and provide access to medical images, pathological slides, genomic data, and clinical records, their utility is often constrained by small sample sizes, inconsistent annotations, and limited accessibility. These limitations reflect broader challenges of data scarcity and uneven distribution in the field. Moving forward, the establishment of high-quality, standardized, and interoperable multimodal databases will be essential to strengthen model generalizability and ensure the safe and effective clinical translation of AI in oral medicine.^124^

Biases in oral datasets undermine representativeness and impede the equitable deployment of AI in clinical practice. Demographic bias reflects the underrepresentation of specific racial, age, and gender groups, as most datasets originate from developed countries, thereby limiting applicability across diverse populations and aggravating health disparities. Geographic bias stems from region-specific data collection, since oral disease prevalence and presentation vary with genetic, environmental, dietary, and healthcare contexts, models trained on single-country datasets may not generalize to other settings.^125^ Socioeconomic bias is driven by unequal access to care, with lower healthcare utilization among disadvantaged groups leading to insufficient representation in training data.^126^ Technical bias arises from heterogeneity in medical devices, imaging protocols, feature selection, and algorithm design, each of which can compromise model accuracy and fairness.

Patient privacy protection remains another key challenge for AI. Effective training and optimization require access to sensitive data, including oral images, medical records, and genetic information. The collection, storage, processing, and sharing of such data carry inherent risks of breaches, unauthorized access, and commercial misuse. With the growing reliance on cloud platforms and cross-institutional data exchange, safeguarding patient privacy has become a pressing ethical concern. To mitigate these risks, privacy requirements must be embedded into AI design from the outset, with approaches such as differential privacy and federated learning providing potential solutions to balance data utility and confidentiality.^127^ Nevertheless, patients often lack sufficient knowledge or control over how their data are used, particularly in commercial contexts, posing significant challenges to the principle of autonomy in medical ethics.

### Intelligent analysis

During data processing, the lack of standardization in oral medical data poses a major obstacle to AI applications. Oral medicine relies on heterogeneous sources—including imaging, biochemical measures, microbiological assays, and behavioural records—collected across varied devices, institutions, and time periods, leading to inconsistent formats and quality. This heterogeneity complicates dataset integration and cross-institutional validation. Annotation practices are also non-uniform, with differences in diagnostic criteria and labelling protocols compounded by noise, sampling bias, and missing information. Overcoming these deficiencies will depend on establishing unified standards for data acquisition, annotation, and interoperability across diverse modalities, paving the way for clinically reliable and widely applicable AI systems.^128^

Besides, key barriers stem from the computational and algorithmic demands of AI systems. Models trained on single dataset often lack generalizability, exhibiting marked performance declines when applied across diverse regions, imaging modalities, or patient populations. Moreover, state-of-the-art architectures contain millions of parameters and require substantial memory and computational power, particularly for high-resolution medical imaging. Hardware limitations further constrain clinical deployment: while graphics processing units (GPUs) accelerate model inference compared with traditional central processing units (CPUs), they come at the cost of high energy consumption and financial burden. Finally, the tension between real-time analysis and diagnostic accuracy persists; achieving rapid inference frequently necessitates model simplification or reduced input resolution, potentially compromising clinical reliability.^129^

### Clinical output

AI systems require regulatory approval for clinical use, with validation standards exceeding those typically applied in academic research. The FDA classifies AI-based medical devices by patient risk, with many designated as moderate risk, for which randomized controlled trials are generally not required. Nevertheless, robust clinical evaluation remains essential for the widespread adoption of AI in clinical settings. While most AI products demonstrate strong task-specific performance, such as disease detection, they often lack generalizability across diverse populations, necessitating further validation. Consequently, only a limited number of AI algorithms are currently approved for clinical deployment. Among those approved for oral medicine, only a few related to HNSCC have been authorized, with primary applications in radiation therapy planning (Table S3).

Clinical integration of AI in oral medicine faces multiple barriers spanning technical, educational, legal, and ethical domains. On one side, AI systems demand high-performance computing infrastructure, stable network connectivity, and large-scale data storage and processing capacity—requirements often beyond the reach of small and medium-sized dental clinics. Implementation also necessitates ongoing system maintenance, upgrades, and professional technical support, further increasing financial and operational complexity. In parallel, successful adoption requires training oral healthcare professionals to develop the technical literacy and operational competence needed for AI-assisted workflows, yet many practitioners have limited exposure to such technologies. This training requires time, resources, and adaptation to new work habits and decision-making paradigms.^130^

Trustworthiness of AI systems poses another critical challenge. The 'black box' nature of many algorithms undermines physicians’ confidence, as poor explainability limits their willingness to rely on AI-generated diagnostic recommendations. Concerns regarding responsibility attribution further complicate adoption: medical professionals remain uncertain about liability when AI errors lead to patient harm, especially amid ongoing debates over data security and privacy protections.^131^ Moreover, the paucity of large-scale randomized controlled trials and long-term follow-up studies constrains evidence-based validation, reinforcing scepticism about the safety and efficacy of AI in oral medicine. Finally, both clinicians and patients express concern that increasing reliance on AI may erode the humanistic aspects of oral healthcare and disrupt traditional doctor-patient relationships, further dampening acceptance.^132^

### Future perspectives

The ongoing development of AI has introduced new possibilities for oral medicine, but its current progress should be interpreted with caution rather than as evidence of imminent clinical transformation. Although many studies report encouraging results, much of the existing evidence remains based on retrospective, single-centre, and proof-of-concept research, and therefore does not yet establish readiness for routine clinical use.^133^ In this context, continued advances in algorithmic efficiency, multimodal data integration, and computational scalability may broaden the technical scope of AI applications, while parallel developments in imaging, multi-omics, and molecular biology may further refine model performance. However, the future value of AI in oral medicine will depend not only on technical improvement, but also on whether these systems can demonstrate reproducibility, generalizability, interpretability, and practical feasibility in real-world clinical settings.

This translational gap is closely related to several unresolved structural limitations. Data resources are still distributed unevenly across disease categories, with oral cancer being relatively well represented, whereas many oral mucosal diseases and rare conditions remain underrepresented. In addition, many currently reported models are developed using retrospective single-centre datasets, raising concerns about robustness across different geographic regions, patient populations, imaging devices, and clinical settings. These limitations suggest that future research should place greater emphasis on multi-centre collaboration, standardized data collection, external validation, and prospective evaluation.

Clinical translation is also constrained by regulatory, practical, and interpretive challenges. To date, AI tools with clearer pathways to implementation have generally emerged in more standardized tasks, whereas diagnostic applications in oral medicine remain less mature. At the same time, the limited explainability of many models, unresolved questions regarding responsibility in AI-assisted decision-making, and the financial and technical barriers faced by smaller clinics continue to restrict broader deployment. Future progress will therefore depend not only on model performance, but also on transparency, governance, workflow integration, and realistic assessments of cost and feasibility.

Multimodal AI may offer a useful direction for future study, particularly through the integration of oral imaging, pathology, clinical records, laboratory findings, and molecular data. Such approaches may improve the characterization of oral disease and, potentially, support broader assessment of systemic health risks. However, these possibilities remain dependent on improvements in data harmonization, handling of missing modalities, interpretability, and prospective validation. Overall, AI in oral medicine should still be regarded as an evolving area with considerable potential, but also with important unresolved limitations that must be addressed before wider clinical use can be justified.

## Author contributions

*Writing - review & editing*: All authors.

*Methodology*: Xinjian Ye, Tingfeng Zha, Xun Xie, Yi Xu, Li Mei, Rui Zhang, Zhiyong Wang.

*Investigation*: Xinjian Ye, Tingfeng Zha, Xun Xie, Yi Xu, Li Mei, Rui Zhang, Zhiyong Wang.

*Conceptualization*: Xinjian Ye, Tingfeng Zha, Xun Xie, Yi Xu, Li Mei, Rui Zhang, Qianming Chen.

*Writing - original draft*: Xinjian Ye, Tingfeng Zha.

*Supervision*: Zhiyong Wang, Qianming Chen.

*Project administration*: Qianming Chen.

*Funding acquisition*: Qianming Chen.

## Funding

This work was supported by the 10.13039/501100001809National Natural Science Foundation of China (No. 82370952, 82330029), National Key R&D Program of China (No. 2022YFC2402900), 10.13039/501100008990Department of Science and Technology of Zhejiang Province (No. 2024ZY01032, 2024C03193).

## Declaration of generative AI and AI-assisted technologies in the manuscript preparation process

During the preparation of this work, the authors used ChatGPT for language polishing. After using this tool, the authors reviewed and edited the content as needed and take full responsibility for the content of the published article.

## Data availability statement

Original data generated and analysed during this study are included in this published article or in the supplementary material.

## Conflict of interest

None disclosed.

## Appendix Supplementary materials

Image, application 1

## Footnotes

Supplementary material associated with this article can be found in the online version at doi:10.1016/j.identj.2026.109798.
