# Supplementary material for: Artificial Intelligence Meets Oral Medicine: Extending the Capabilities of Human Intelligence
Source: Int Dent J. 2026 Aug 5;76(5):109798. doi: 10.1016/j.identj.2026.109798 (PMC13470172; doi:10.1016/j.identj.2026.109798)
Supplement: Supplementary file 1 [file mmc1.docx]

**Table S1** Representative studies of AI applications in oral medicine.

| **Study** | **Application** | **Disease** | **Input data** | **AI model** | **Sample size** | **Metrics** |
| --- | --- | --- | --- | --- | --- | --- |
| (Zhou et al, 2024) | Lesion classification and detection | RAU | Intraoral photographs | 7 CCNs | Training (628), validation (78), testing (79) | Precision (93%), recall (92%),  F1 score (92%), sensitivity (92%), AUC (0.99) |
| (Wu et al, 2024) | Automatic diagnosis | Sjögren’s syndrome | WSI | CTG-PAM | Training (36), external testing (64) | Accuracy (94%), sensitivity (98%), AUC (0.80) |
| (Yuan et al, 2023) | Automatic diagnosis | OSCC | OCT images | MDRL | Training (346), testing(122) | Precision (85%), recall (91%), sensitivity (91%),  AUC (0.92) |
| (Wang et al, 2025) | Image enhancement | OPMDs | Intraoral photographs | YOLO | Training (6488),  validation (2592), testing (328) | Accuracy (91-97%),  F1-score (70-91%),  sensitivity (62-86%) |
| (Grinberg et al, 2025) | Differential diagnosis | Oral mucosal lesions | Clinical data | ChatGPT | 100 | High sensitivity for malignant lesions |
| (Vueghs et al, 2024) | Automatic diagnosis | Orofacial pain | Clinical data | ChatGPT | 100 | Overall diagnostic performance (52%), differential diagnosis quality (80%) |
| (Shephard et al, 2024) | Risk assessment | Oral epithelial dysplasia | WSI | HoVer-Net, MLP | Training (193), external validation (89) | Recall (92%),  F1-score (69%),  sensitivity (92%),  AUC (0.75) |
| (Zhi et al, 2025) | Auxiliary diagnosis | HNSCC | 16S rRNA amplicon sequencing, metagenomic sequencing | Random forest | Training(120),  testing(52) | AUC (0.78-0.89) |
| (Zeng et al, 2024) | Auxiliary diagnosis | Persistent pulmonary nodules | Saliva 16S rRNA sequencing | LightGBM | Training (154),  testing (66) | AUC (0.88) |
| (Mei et al, 2025) | Prognosis evaluation | OSCC | Clinical data | Logistic regression | Training (563),  validation (600) | F1-score (41%),  sensitivity (68%),  AUC (0.72) |
| (Song et al, 2025) | Prognosis evaluation | HPV-associated OSCC | CT, WSI | SMuRF | Training (120),  validation (52),  testing (105) | AUC (0.74) |
| (Yao et al, 2025) | Recurrence evaluation | HNC | Clinical data | LASSO, random forest | 10-fold cross validation (1302) | Recall (83%), sensitivity (83%), AUC (0.68),  specificity (58%) |
| (Liu, Zhang, et al, 2024) | Metastasis predicting | OTSCC | MRI | KNN | Training (89),  testing (39) | Sensitivity (67%), specificity (80%), AUC (0.87) |
| (Wang et al, 2024) | Therapeutic response prediction | OLK | WSI | Cox regression | Training (30),  validation (139) | Accuracy (84%),  AUC (0.88) |
| (Alabi et al, 2023) | Complication prediction | HNC | CT | Random forest | Training (39),  testing (10) | Accuracy (90%), precision (100%), F1 score (91%),  sensitivity (83%),  AUC (0.92) |
| (Yang *et al*, 2024) | Radiotherapy plan | HNC | CT | Deep learning | Training (20), testing (100) | High efficiency and quality |
| (Yoo and Sim, 2022) | Surgical navigation | NA | CT | Machine learning | NA | TRE (0.77 ± 0.13 mm), SRE (0.93 ± 0.17 mm) |
| (Boehm et al, 2025) | Intraoperative margin assessment | HNC | HSI | GNN | 32 patients, 59 HSI | Accuracy (89%),  F1-score (85%),  AUC (0.95) |
| (Salama et al, 2025) | Pain prediction | OCC/OPC | Clinical text | Gradient boosting machine | Training (587), testing (251) | Precision (86%), recall (39%),  F1-score (48%),  AUC (0.71) |

AUC, area under the receiver operating characteristic curve; CNN, convolutional neural network; ConvNeXt, convolutional network next; CT, computed tomography; CTG-PAM, cross-task guided progressive attention model; DFS, disease-free survival; GNN, graph neural network; GPT, generative pre-trained transformer; HNC, head and neck cancer; HNSCC, head and neck squamous cell carcinoma; HoVer-Net, horizontal and vertical network; HPV, human papillomavirus; HSI, hyperspectral imaging; KNN, k-nearest neighbor; LASSO, least absolute shrinkage and selection operator; LightGBM, light gradient boosting machine; MDRL, multi-level deep residual learning; MLP, multilayer perceptron; MRI, magnetic resonance imaging; OCC, oral cavity cancer; OCT, optical coherence tomography; OLK, oral leukoplakia; OPC, oropharyngeal cancer; OPMDs, oral potentially malignant disorders; OPSCC, oropharyngeal squamous cell carcinoma; OS, overall survival; OSCC, oral squamous cell carcinoma; OTSCC, oral tongue squamous cell carcinoma; RAU, recurrent aphthous ulcers; SMuRF, Swin Transformer-based multimodal and multi-region fusion framework; SRE, surface registration error; SUS, system usability scale; TRE, target registration error; WSI, whole slide image; YOLO, You Only Look Once.

**Table S2** Publicly available datasets in oral medicine.

| **Dataset** | **Details on Dataset** | **Cons** | **Pros** | **Restrictions in Data Sharing** | **Access Link** |
| --- | --- | --- | --- | --- | --- |
| ORCHID | A database integrating high-resolution case images of various oral cancers and precancerous lesions, including 150 cases. | - Limited field of view - High computational resource requirements - Annotation work is complex and prone to inconsistencies - Poor model generalization ability | - A large number of high-resolution oral pathology images - High dataset quality and reliability - Promotes accurate AI diagnosis of oral diseases | Open access | https://zenodo.org/records/12636426 |
| TCGA-HNSC | The HNSCC dataset from the Cancer Genome Atlas (TCGA), including genomic data and imaging data. | - Complex preprocessing required - High dimensionality of genomic data - Limited imaging modalities  - Requires integration of genomic and imaging data | - Integrates genomic and imaging data - Supports survival analysis and outcome research | -Requires agreement to TCGA's data-use policy  -Certain portions require dbGaP application | portal.gdc.cancer.gov/projects/TCGA-HNSC |
| TCIA-HNSCC | The HNSCC dataset in The Cancer Imaging Archive (TCIA) includes CT/PET images and clinical data of screening participants, comprising 627 cases. | -Some data is missing -There are inconsistencies in the data | -Combining imaging data with clinical information -The data exhibits high consistency and reliability | -Adhering to the NIH Controlled Data Access Policy | https://www.cancerimagingarchive.net/collection/hnscc/ |
| Comprehensive endoscopic HSI dataset | A large-scale, annotated endoscopic hyperspectral imaging oral dataset, containing 226 cases. | -The data may not reflect real clinical conditions -The diversity of patients is limited. | -Widely applicable. -High annotation quality. | Permission from the corresponding author is required | https://medinform.jmir.org/2025/1/e76148 |
| MODID | A multispectral imaging dataset containing data from 91 patients with oral diseases. | - Small sample size. - Limited patient diversity. - Complexity of data preprocessing. | -High quality segmentation annotations -Suitable for image segmentation research | Open access | https://datadryad.org/dataset/doi:10.5061/dryad.nvx0k6dxw |
| Dataset of Annotated Oral Cavity Images for Oral Cancer Detection | A large-scale dataset of oral images captured by  mobile phones, containing 716 cases, along with demographic information. | - Some images have poor resolution - Annotation quality is limited | - Large-scale and diverse - Enables early disease diagnosis research | Consent from the publisher is required | https://zenodo.org/records/10664056 |
| NDB-UFES | A dataset of oral cancer and leukoplakia, consisting of histopathological images and demographic data, involving 69 patients. | - Small sample size - Some data is missing | - Rich disease diversity - Enables disease diagnosis research | Open access | https://data.mendeley.com/datasets/bbmmm4wgr8/4 |
| HANCOCK | A multimodal dataset on head and neck cancer, containing data from 763 patients. | - Some data is missing - Annotations are sparse - Text data has not been fully explored | - A multimodal dataset - Large-scale | Open access | https://hancock.research.fau.eu/ |
| RADCURE | A CT dataset integrating demographic, clinical, and treatment information of head and neck cancer, containing 3,346 cases. | - Staging based on AJCC 7th edition - Some demographic and clinical information is missing | - Large-scale. - Provides longitudinal data - Enables radiomics research | Adhering to the NIH Controlled Data Access Policy | https://www.cancerimagingarchive.net/collection/radcure/ |
| AAPM-RT-MAC | A T2-weighted MRI dataset of head and neck cancer patients, containing 55 cases. | - Small sample size and limited diversity - Does not include tumor contours - Lacks demographic and other information - Focuses solely on segmentation tasks | - Suitable for radiotherapy research - High data reliability - Enables radiomics research | Adhering to the NIH Controlled Data Access Policy | https://www.cancerimagingarchive.net/collection/aapm-rt-mac/ |
| RTOG 0522 | A dataset containing CT images and radiotherapy plans of stage III and IV head and neck cancer patients, involving 891 cases. | - Some cases lack radiotherapy data | - Large-scale - Provides longitudinal data - Enables radiomics research | Adhering to the NIH Controlled Data Access Policy | https://www.cancerimagingarchive.net/collection/head-neck-cetuximab/ |
| CPTAC-HNSCC | A head and neck cancer dataset containing histopathological images, whole slide images, CT, MRI, and PET data, consisting of 207 cases. | - High dimensionality of genomic and radiomics features - Requires complex preprocessing | - Integrates genomic data with imaging data - Enables survival analysis and outcome research | Adhering to the NIH Controlled Data Access Policy | https://www.cancerimagingarchive.net/collection/cptac-hnscc/ |
| HEAD-NECK-RADIOMICS-HN1 | A dataset containing clinical data and CT scans of 137 HNSCC patients who underwent radiotherapy. | - Differences in imaging quality. - Variability in preprocessing. | - Enables radiomics research - Provides longitudinal data | Adhering to the NIH Controlled Data Access Policy | https://www.cancerimagingarchive.net/collection/head-neck-radiomics-hn1/ |
| QIN-HEADNECK | A dataset based on PET/CT scan images of head and neck patients, along with relevant clinical information, involving 156 cases. | - Limited data scale - Clinical data is not fully standardized. - High variability in annotations. | - Provides longitudinal data - High-quality segmentation annotations | Adhering to the NIH Controlled Data Access Policy | https://www.cancerimagingarchive.net/collection/qin-headneck/ |
| BD2Decide | A radiomics analysis dataset based on MRI for head and neck cancer, containing 519 cases. | - Some patients are missing certain MRI sequences - MRI data has center bias - Image variability | -Multi-center, Multi-location -Large sample size | Permission must be obtained from the relevant research institution | https://www.thegreenjournal.com/article/S0167-8140(23)00176-7/fulltext |

AJCC, American joint committee on cancer; CT, computed tomography; HNSCC, head and neck squamous cell carcinoma; MRI, magnetic resonance imaging; NIH, national institutes of health; PET, positron emission tomography; TCGA, the cancer genome atlas; TCIA, the cancer imaging archive.

**Table S3** FDA approved AI devices in oral medicine.

| **Name** | **Data modality** | **Task** | **FDA summary** | **Year** |
| --- | --- | --- | --- | --- |
| AccuContour | CT | Segment CT images and automatically generate radiotherapy plans | https://www.accessdata.fda.gov/cdrh_docs/pdf22/K221706.pdf | 2023 |
| AI Segmentation | CT | Segment the head and neck anatomical structures for radiotherapy planning | https://www.accessdata.fda.gov/cdrh_docs/pdf21/K211881.pdf | 2021 |
| Auto Segmentation | CT | Segment the head and neck anatomical structures for radiotherapy planning | https://www.accessdata.fda.gov/cdrh_docs/pdf23/K230082.pdf | 2023 |
| Automatic Anatomy Recognition | CT | Segment the head and neck anatomical structures for radiotherapy planning | https://www.accessdata.fda.gov/cdrh_docs/pdf20/K203610.pdf | 2021 |
| Contour+ (MVision Al Segmentation) | CT、MRI | Outline the regions of interest in the head and neck for radiotherapy planning | https://www.accessdata.fda.gov/cdrh_docs/pdf24/K241490.pdf | 2024 |
| INTContour | CT | Segment the head and neck anatomical structures for radiotherapy planning | https://www.accessdata.fda.gov/cdrh_docs/pdf21/K212274.pdf | 2022 |
| Oncospace | CT、MRI | Assist in developing radiotherapy plans for head and neck cancers | https://www.accessdata.fda.gov/cdrh_docs/pdf24/K242748.pdf | 2025 |
| Radiation Planning Assistant | CT | Automatic delineation of head and neck tumors and planning of radiotherapy treatments | https://www.accessdata.fda.gov/cdrh_docs/pdf22/K222728.pdf | 2023 |
| RT-Mind-AI | CT | Segment the head and neck anatomical structures for radiotherapy planning | https://www.accessdata.fda.gov/cdrh_docs/pdf21/K213155.pdf | 2021 |

AI, artificial intelligence; CT, computed tomography; FDA, food and drug administration; MRI, magnetic resonance imaging; PET, positron emission tomography.
